# Age-Related Quantitative and Qualitative Changes in Decision Making Ability

**DOI:** 10.1155/2008/893727

**Published:** 2008-04-11

**Authors:** Valeria Isella, Cristina Mapelli, Nadia Morielli, Oriana Pelati, Massimo Franceschi, Ildebrando Marco Appollonio

**Affiliations:** ^1^Neuropsychology UnitNeurology SectionS. Gerardo HospitalDepartment of NeuroscienceUniversity of Milano BicoccaMonzaItaly; ^2^Neurology DepartmentIRCCS MultimedicaCastellanzaItaly

**Keywords:** Decision making, gambling task, aging, frontotemporal dementia

## Abstract

The “frontal aging hypothesis” predicts that brain senescence affects predominantly the prefrontal regions. Preliminary evidence has recently been gathered in favour of an age-related change in a typically frontal process, i.e. decision making, using the Iowa Gambling Task (IGT), but overall findings have been conflicting. Following the traditional scoring method, coupled with a qualitative analysis, in the present study we compared IGT performance of 40 young (mean age: 27.9 ± 4.7) and 40 old (mean age: 65.4 ± 8.6) healthy adults and of 18 patients affected by frontal lobe dementia of mild severity (mean age: 65.1 ± 7.4, mean MMSE score: 24.1 ± 3.9). Quantitative findings support the notion that decision making ability declines with age; moreover, it approximates the impairment observed in executive dysfunction due to neurodegeneration. Results of the qualitative analysis did not reach statistical significance for the motivational and learning decision making components considered, but approached significance for the attentional component for elderly versus young normals, suggesting a possible decrease in the ability to maintain sustained attention during complex and prolonged tasks as the putative deficit underlying impaired decision making in normal aging.

## 1. Introduction

The 'frontal aging hypothesis' [11] claims that brain senescence affects predominantly the frontal lobes. Provided that the ventromedial prefrontal cortex represents the putative neural substrate of decision making ability [4], an impairment in the ability to decide competently might be an important, life-relevant neuropsychological correlate of physiological aging.

Recently, decision making competency in healthy elderly has been the subject of several studies [5–7, 12], which used a classical decision making test, the Iowa Gambling Task (IGT) [1]. Overall, findings with respect to the effects of aging have been conflicting.

In addition to the traditional scoring methods of the IGT, a qualitative analysis has been devised lately [3], which allows to decompose the cognitive processes underlying the task according to an *expectancy-valence learning* model of decision making [2]. The analysis considers motivational, memory and attentional components and has been validated in a number of clinical populations [13].

In the present survey we replicated the design of the aforementioned studies, i.e. comparison of performance of young and old normal individuals at the Iowa Gambling Task, while adding the following: 1. a group of patients affected by mild frontotemporal dementia – frontal variant, which may be considered as the pathological counterpart of frontal aging, and 2. qualitative analysis of performance at the IGT. Our aim was to try answering to the following questions: are healthy elderly as good as young individuals at making decisions? If not, are they comparable to mild frontal

ISSN 0953-4180/08/$17.00 © 2008 – IOS Press and the authors. All rights reserved dementia patients, i.e. do they present an equal level of impairment and similar changes in decision making heuristic?

## 2. Subjects and methods

Normal participants were 80 community dwelling, healthy individuals with a MiniMental State Examination (MMSE) adjusted score $\geqslant$ 24; 40 young controls aged 20 to 35 and 40 elderly controls aged 50 or more. Demented participants were 18 subjects selected among outpatients referring to the Neuropsychology Services at S. Gerardo Hospital, in Monza, and Clinica Santa Maria, in Castellanza, Milan, and meeting standardised diagnostic criteria for frontal variant of fronto-temporal dementia [8]. Normals and patients were excluded if they had a current or past history of major psychiatric disorders (including depression), head trauma or alcohol or drug abuse. Only patients with a MMSE adjusted score $\geqslant$18 or a Clinical Dementia Rating score between 0.5 and 2 were included. Six frontal patients out of 18 (33.3%) had a CDR score of 0.5, seven (38.9%) had 1.0 and five (27.8%) had 2.0. The socio-demographic features and MMSE scores of the three study groups are shown in Table 1. As expected, MMSE scores were significantly worse for demented subjects, while they were overlapping for the two control groups. Although schooling was significantly higher for young controls than for the other two groups, we did not covariate for education when analysing experimental data, because educational level appeared to be highly correlated with age (Pearsons' r coefficient = 0.60), so that covarying would have masked possible intergroup age-related differences. Besides, the total IGT score was unrelated with education (maximum Pearson's r coefficient = 0.36 – obtained in the elderly controls' group). The possibility that statistically significant differences might have been biased should anyway be taken into account.

All demented subjects underwent a standard assessment including neurological, neuropsychological and neuroimaging examinations. Normal controls underwent the MMSE and a semistructured clinical interview for the verification of exclusion criteria.

The manual version of the Iowa Gambling Task was administered by two neuropsychology trainees according to the instructions provided by Authors [1]. Briefly, the subject receives a budget of €4000 (facsimile bills) and is invited to choose one card at a time from four decks (A to D), for 100 trials. The four decks are differently arranged in terms of amount of wins, and frequency and amount of losses, so that A and B are disadvantageous decks (i.e. lead to net financial loss), while C and D are advantageous decks (i.e. lead to net financial gain). The subject must try to win as much money as possible, and, at the same time, to avoid losing money as far as possible.

Quantitative IGT parameters consist in the net number of advantageous choices (selections from decks C and D minus selections from decks A and B), computed both for the whole 100 cards (total score) and for five successive blocks of 20 cards each (1–20, 21–40 and so on); this latter parameter is used in order to quantify the progressive change in selection across the task.

Qualitative analysis [3] was performed for each participant with the original Matlab algorithm provided online by authors (http://mypage.iu.edu/~jbusemey/home.html) and considers three parameters, each corresponding to a putative cognitive component of the decision making process. The *motivational* parameter refers to the relevance attributed to wins; it ranges from 0 to 1 and higher values indicate higher relevance. The *learning* parameter refers to memory for the outcome of prior choices; it ranges from 0 to 1 and higher values indicate more rapid forgetting. The *sensitivity* parameter refers to the sensitivity of choices to expectancies progressively formulated during the test and is deemed to depend on the level of concentration maintained throughout the task; it ranges from $-5$ to $+5$ and higher values indicate higher levels of concentration.

Statistical analysis was performed using SPSS 14.0. Demographic and neuropsychological variables were analysed using chi-square test or one-way ANOVA, as appropriate. Results of the Iowa Gambling Task were also analysed using a repeated measures ANOVA design, with card blocks [1–5] as within-subjects and group (young controls vs elderly controls vs frontal patients) as between-subjects variables. Besides, each participant was categorized as "good" or "poor" performer at the IGT, based on whether he/she made a significantly greater number of advantageous than disadvantageous choices (using the binomial test [9]); the proportion of good and poor performers within each study group was then compared. All tests were thresholded at a significance level of $p < 0.05$.

## 3. Results

The comparison among the three study groups for IGT scores are shown in Table 1.

Table 1
Sociodemographic and neuropsychological features and gambling task parameters of the three study groups

| Variables | Young controls $n = 40$ | Elderly controls $n = 40$ | Frontal dementia patients $n = 18$ | F value/x2 |
|---|---|---|---|---|
| | MEAN (SD) | MEAN (SD) | MEAN (SD) | |
| Age | 27.9 (4.7)*° | 65.4 (8.6) | 65.1 (7.4) | 333.6 |
| Gender (% men) | 38 | 45 | 56 | 1.7 |
| Education (years) | 13.1 (2.1)*° | 8.7 (3.6) | 7.5 (4.5) | 26.1 |
| Adjusted MMSE score | 29.0 (1.5)° | 28.9 (0.9)° | 24.1 (3.9) | 44.7 |
| | MEAN (SEM) | MEAN (SEM) | MEAN (SEM) | |
| IGT total score | 14.2 (3.8)° | 7.1 (3.1) | −2.2 (3.9) | 3.9 |
| Poor performers at the IGT (%) | 57.5*° | 80 | 88.9 | 8.1 |
| IGT qualitative parameters: | | | | |
| Motivation | 0.47 (0.1) | 0.62 (0.1) | 0.44 (0.1) | 1.9 |
| Learning | 0.21 (0.1) | 0.36 (0.1) | 0.36 (0.1) | 2.0 |
| Sensitivity | 0.47 (0.3) | −0.91 (0.4) | −0.68 (0.8) | 2.9 |

*$p < 0.05$ vs elderly controls, °$p < 0.05$ vs frontal dementia patients.
LEGEND: SD = Standard Deviation, SEM = Standard Error of the Mean, MMSE = MiniMental State Examination, CDR = Clinical Dementia Rating, IGT = Iowa Gambling Task.

Overall, one-way ANOVA for the total scores was significant and post-hoc tests showed a significantly lower score for demented subjects with respect to young normals and an intermediate score, not significantly different from that of the other two groups, for elderly controls.

Repeated measures 3 (group) x 5 (block) ANOVA (Fig. 1) showed significant effects for block (F(4,380) = 7.133, $p = 0.000$), group (F(2,95) = 3.934, $p = 0.023$) and block x group interaction (F(8,380) = 3.198, $p = 0.002$). Post-hoc analysis revealed that demented subjects showed a flat IGT score curve across blocks; young subjects chose significantly more advantageously than elderly subjects at the last block and than frontal patients at the last three blocks; elderly controls chose significantly more advantageously than frontal patients at the fourth block only.

Chi-square comparison of the proportion of good and poor performers showed a significantly higher percent of good performers within the young control group with respect to the other two groups, which did not differ from each other.

One-way ANOVA comparison of the three qualitative parameters was not significant for the motivational and learning parameters and approached significance for the sensitivity parameter ($p = 0.056$); at post-hoc comparisons, sensitivity was lower for elderly normals with respect to young normals ($p = 0.021$).

## 4. Discussion

In the present study we compared the performance at a gambling task of young normals, elderly normals and patients with frontal neurodegeneration, with the aim to evaluate possible quantitative and qualitative changes in decision making ability related to frontal aging.

Previous literature dealing with the effects of aging on decision making as assessed via the IGT has yielded controversial results. Wood et al. [12] evidenced a different cognitive strategic approach to the task for young and old healthy adults, but found no difference in their overall quantitative performance. MacPherson et al. [7] as well reported no adverse effect of aging on successful completion of the task (although the fact that their young participants performed rather poorly might have been a confounding factor). Conversely, Denburg et al. [5,6] found an age-related decline in decision making ability as assessed with the IGT. Such a decline concerned a substantial minority of their normal seniors and appeared to be unrelated to their sociodemographic, general health or neuropsychological status.

Our findings are in line with these latter studies. IGT total scores did not differ significantly among the three study groups, probably due to large variances, but subgroup analysis revealed a significantly major proportion of poor performers among elderly normals and frontal patients. More precisely, the three study groups were substantially overlapping at the beginning of the task; from the second to the fourth block, a progressive increase in the number of advantageous choices took place for both control groups, not for demented patients; in the final block, young normals went on doing profitable selections, while seniors started to make a higher and higher number of disadvantageous choices and dropped down almost to the level of demented patients. As already stated in the methods section, educa-

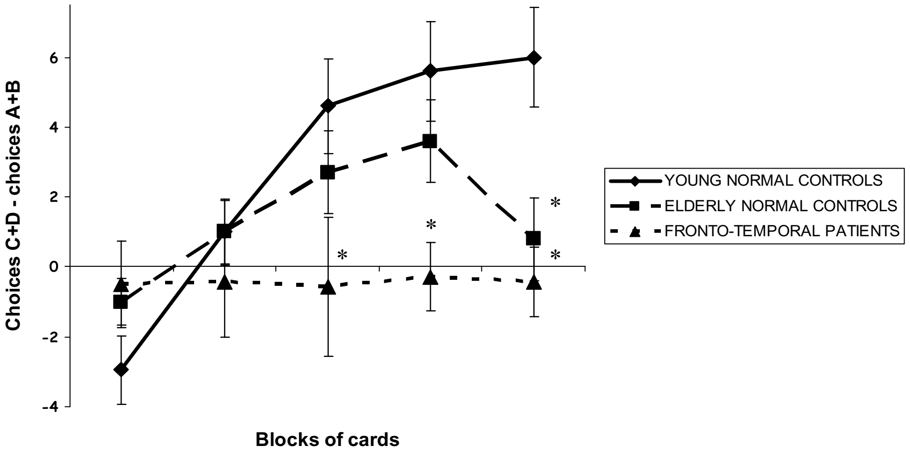

*p<0.05 vs young controls, p<0.05 vs elderly controls

Fig. 1. Performance of the three study groups at the Iowa Gambling Task, with each block including 20 successive cards. The score is represented by the number of choices from advantageous decks (C and D) minus the number of choices from disadvantageous decks (A and B). A negative score indicates poor decision making.

tional level was significantly higher for young than for old participants, but covarying was not carried out because it might have masked age-related discrepancies. Albeit a bias might still have derived from such a difference, we do not deem it sufficiently large to change our conclusions.

According to qualitative findings, the elderly controls' behaviour did not seem to be due to an alteration of the process of weighing positive feedbacks, neither to a defective learning of expected outcomes associated with the different choice options. Rather, it might have been related to some factor, most likely a progressive loss of concentration, which interfered with the consistent application of cards selection strategy followed by the subject during previous trials, leading to more erratic choices in the final blocks of cards. In fact, healthy seniors presented lower values of the sensitivity parameter with respect to young normals. Such a result, however, approached, but did not reach significance, and thus requires confirmation. The specificity of this 'attentional' component to decision making would also warrant investigation.

As to frontal demented individuals, no qualitative parameter was able to explain failure at the IGT, including sensitivity. However, an attentional deficit might still be a possible account for frontal patients' performance, albeit not detected by the sensitivity parameter. As a matter of fact, sensitivity is related to a *progressive* loss of concentration towards the end of the task and would not signal a random pattern of choices possibly set up from the beginning of the test.

To the best of our knowledge, there is only one study that used Busemeyer's algorithm in order to examine the qualitative features of performance of old and young normals at the IGT, yielding partially different results compared to the present ones [12]. In Woods et al.'s studies two groups of subjects were equally successful at the task. Secondly, they appeared to apply different strategies: young participants placed more emphasis on memory, while a more proper appraisal of positive and negative feedbacks represented seniors' strength (differences in the sensitivity parameter could not be evaluated independently from those obtained in the learning parameter). Dissimilarities in participants' features possibly affecting the approach to the task, like age and educational level (which were both higher in Wood et al.'s elderly sample), or the use of the computerized, rather than the manual, version of the test might perhaps partly account for such discrepancies, but mostly they remain unexplained. Likewise, the present results can be hardly reconciled with those of the one prior study dealing with decision making ability in the frontal variant of fronto-temporal dementia [10], where age-matched controls largely outperformed patients at the IGT.

Overall, our findings suggest that healthy seniors undergo a decline in their ability to decide advantageously, performing at the IGT at an intermediate level with respect to young normals and patients with frontal dysfunction due to neurodegenerative pathology. The putative deficit underlying such a decline might involve the capacity to maintain sustained attention during complex and prolonged decision making tasks. The lack of straightforward data in support for this hypothesis and discrepancies with respect to previous literature data, however, prompt further investigation of the issue.
